# Lacosamide in the general population and in people with intellectual disability: Similar responses?

**DOI:** 10.1016/j.seizure.2020.02.013

**Published:** 2020-03

**Authors:** Jon Allard, William Henley, Brendan Mclean, Adrian Sellers, Sharon Hudson, Sanjeev Rajakulendran, Adrian Pace, Sarah Pashley, Melissa Maguire, Monica Mohan, Shan Ellawela, Phil Tittensor, Sunil Ram, Manny Bagary, Rohit Shankar

**Affiliations:** a Cornwall Partnership NHS Foundation Trust, United Kingdom; b University of Exeter Medical School, United Kingdom; c Royal Cornwall Hospital NHS Foundation Trust, United Kingdom; d National Centre for Neurology and Neurosurgery, University College Hospitals NHS Foundation Trust, United Kingdom; e Salford Royal NHS Foundation Trust, United Kingdom; f Nottinghamshire Healthcare NHS Foundation Trust, United Kingdom; g Leeds Teaching Hospitals NHS Trusts, United Kingdom; h North Bristol NHS Trust, United Kingdom; i The Newcastle upon Tyne Hospitals NHS Foundation Trust, United Kingdom; j The Royal Wolverhampton NHS Trust, United Kingdom; k Somerset Partnership NHS Foundation Trust, United Kingdom; l Birmingham and Solihull Mental Health NHS Foundation Trust, United Kingdom

**Keywords:** Seizure, Antiseizure drug, Intellectual disability, Neurodevelopment, Developmental disability

## Abstract

**Purpose:**

Epilepsy prevalence is significantly higher in people with Intellectual Disability (ID) compared to people with epilepsy (PWE) from the general population. Increased psychological and behavioural problems, healthcare costs, morbidity, mortality and treatment resistance to antiepileptic drugs (AEDs) is associated with epilepsy in ID populations. Prescribing AEDs for PWE and ID is challenging and influenced heavily by studies conducted with the general population.

Our study compares Lacosamide (LCM) response for the ID population to those from the general population; using data from an UK based epilepsy database register (EP ID/PDD AED Register).

**Methods:**

Pooled retrospective case notes data for PWE prescribed LCM at 11 UK NHS Trusts were analysed. Participants were classified as per WHO guidance into groups of moderate-profound ID, mild ID and General population. Demographics, concomitant AEDs, starting and maximum dosage, exposure length, adverse effects, dropout rates, seizure frequency were collected. Group differences were reported as odds ratios estimated from univariable logistic regression models.

**Results:**

Of 232 consented participants, 156 were from the general population and 76 had ID (24 mild, 52 moderate-profound). Twelve month withdrawal rates and reasons, efficacy, side-effects, start and maximum doses were similar between the groups. Dose titration between baseline and three months was significantly slower in the ID group (p = 0.02).

**Conclusion:**

There were no differences for LCM outcomes between general and ID groups. Slower LCM titration in ID populations in the first 3 months was associated with higher retention and lower behavioural side effects as compared to similar European studies.

## Highlights

• Lacosamide response in Intellectual Disability (ID) is alike to general population.
• Efficacy is comparable to similar studies from the UK, Germany & the Netherlands.
• Higher retention & lower behavioural side-effects noted to Dutch & German studies.
• Titration differs significantly in first three months between ID and non-ID group.
• Findings of the UK Ep-ID Register support the safe use of LCM in the ID population.

## 1 Introduction

Epilepsy prevalence is approximately 0.6–1.0 % across the worldwide general population [1]. It is much higher in the intellectual disability (ID) population, where around 22 % have epilepsy [2]. It is also estimated that as many as one in four people with epilepsy (PWE) have an ID [3]. Whilst seizure control for all PWE is initially managed by licensed Anti-Epileptic Drugs (AEDs), those with ID are excluded from pre-market systemic trials [4]. Rates of treatment resistance are significantly higher in ID populations than general [5]. The scientific evidence base for AEDs is therefore predominantly focussed on PWE from the general population, with limited research on PWE and ID [3].

The clinical picture for PWE and ID is complicated by increased physical and mental health comorbidities [6], with an average of ten other co-morbid health issues present [7]. The presentation of seizures alongside these health issues means accurate diagnosis and AED prescribing is challenging [8]. Increased vulnerability to cognitive and behavioural side effects, but reduced ability to communicate them, further complicate the clinical picture [9,8]. There is also limited evidence around quality of life and epilepsy [10,11] and increased potential for drug resistance and interactions [12,13]. Scientific, anecdotal, peer, carer and personal feedback are all subsequently utilised [13] as clinicians are expected to monitor risks and prescribe with caution [9,8].

Epilepsy has a huge impact on the everyday lives of this complex and vulnerable population, along with the families and carers who help look after them [14]. A person with ID is five times more likely than someone from the general population to have an avoidable emergency attendance, with seizures responsible for nearly half of such attendances [15]. There is recognition of high rates of premature mortality in this vulnerable population with 45 % of all premature deaths in people with ID associated with seizures in the five years prior to demise [16]. The impact of epilepsy drives an ethical and economic debate for building an evidence base to help inform decision making around AED treatment options [17,18,8].

There is also an increasing recognition that the ID population itself is not a homogenous group. Those with mild ID - as recognised by ICD 10/DSM 5 classification [19,20] - are clinically and socially different in their presentation from those with moderate to profound ID. Epilepsy prevalence varies with level of ID, with approximately 10 % of individuals with mild ID and 30 %–50 % of individuals with moderate, severe or profound ID diagnosed with epilepsy [2,21,14]. Comorbidity also varies [6], building an argument for layered guidance around medication use within the ID population. Epilepsy in those with moderate to profound ID is often more severe and treatment resistant than mild ID [5].

We report findings here from one arm (lacosamide) of an UK based database register for PWE and ID [22]. Lacosamide (LCM) is licenced for the adjunctive treatment of treatment resistant partial-onset seizures [23] and subsequently for monotherapy for focal epilepsy [18]. LCM has a novel mode of action as a functionalized amino acid that enhances the slow inactivation of voltage-gated sodium channels without affecting the fast inactivation of voltage-gated sodium channels. This inactivation prevents the channel from opening, helping end the action potential. It has been deemed suitable for prescribing across all PWE, including those with ID.

A systemic review of fourteen predominantly post license studies of LCM in 3509 adult patients, demonstrated seizure frequency reduction of at least 50 % in 18–69 % of patients with refractory focal seizures (across studies) and potential for seizure termination in refractory status epilepsy (evidence from three studies) [24]. LCM was also found to be well tolerated when used in combination with other AEDs. Elsewhere Cawello reported a favourable and predictable pharmacokinetic profile and low potential for drug interactions [25]. Side effects reported for LCM include those common with other AEDs such as dizziness (22.1 %), vision disturbances (10.1 %), headache (6.8 %) and nausea (6.3 %) [24].

Despite this substantive evidence - ranging from large scale prospective RCTs [26] to small scale descriptive retrospective studies [27] - there is limited evidence relating to the use of LCM for PWE and ID. Three European studies (UK, German and Dutch) drawing on retrospective observational data held in clinical records have however been published (Table 1. Western European Studies of LCM within ID populations)

**Table 1:** Western European Studies of LCM within ID populations.

Study	Flores et al 2012 (UK)	Böttcher et al 2017 (Germany)	Brenner et al 2017 (Netherlands)
Cohort	73 Adult ID patients (from wider 403 patient cohort) (Mean age N/K)	136 Adult and Children ID patients (mean age 32.7)	132 Adult ID patients (mean age 41.7)
ID type	Unknown	48 % Mild52 % Moderate -Severe	24 % Mild76 % Moderate-Severe
Setting	Outpatients (UK)	Outpatients and Inpatients (Germany)	Inpatients (Netherlands)
Retention (12 months)	63 %	62 %	64 %
Mean dose	252 mgs	410 mgs	243.6 mgs
Efficacy / Seizure reduction	> 50 % in 32 % of patients (recorded at final follow up)	Not recorded	49 % (undefined) seizure improvement
Reported adverse effects (mental health/behavioural)	48.7 % (behavioural side effects rare)	19 % (unclear)	63 % (Behavioural side effects 30 %)

The UK study draws data from 19 UK NHS Trusts which had a cohort of PWE and ID (n73) within the wider study population [28]. Findings support the safe and effective use of LCM as an adjunctive therapy in partial-onset seizures for those with ID. They did however have a slightly lower but not significantly different response rate to LCM, for people with ID along with less adverse effects and slightly higher rates of withdrawal, whilst behavioural side effects were rare or absent [28].

The German study was focused on three years of retention rates of LCM of 136 adults and children with epilepsy and ID from a single centre [18]. Retention rates for the first year were similar to that reported by the UK, whilst adverse events were less reported. It is worth noting that one third of participants pre-study were recognised to have behavioural issues. No description on effectiveness was provided.

A third similar sized study (n 132) reported on PWE and ID from the adult population living in three specialised care facilities in the Netherlands [4]. Retention rates within 12 months were similar to the UK and German studies. While there was nearly 50 % effectiveness, adverse effects of LCM were noted in nearly two third (62.9 %) of participants with one third being behavioural side effects (30 %). Whilst not dependent on severity of ID, adverse effects were far more prominent than found in general population studies, whilst physical side effects were less reported. All studies found concomitant use of LCM with a sodium channel blocker AED did not influence retention.

## 2 Methods

‘A register for collecting and measuring outcomes of licensed Anti-Epileptic Drugs in patients with Epilepsy and Intellectual Disability and/or Pervasive Developmental Disorders (PDD) ‘Cornwall EP ID/PDD AED Register’

The data presented in this paper has been drawn from the Cornwall EP ID/PDD AED Register, a UK NHS based database register for PWE who have an ID and/or a Pervasive Developmental Disorder PDD. The database register has NHS ethics approval reference: 14/SC/1270 and has been adopted as part of the UK National Institute of Health Research NIHR 31484 portfolio. The data in this paper focusses on the LCM arm exclusively. Further details of the EP ID/PDD Register can be found in Appendix 1.

### 2.1 Consent

Adults aged over 18 and with an epilepsy diagnosis who had been prescribed LCM (currently taking or withdrawn) at any point prior to 18/12/2017 were approached at eleven UK collaborating sites (one lead site and 10 NHS Trusts acting as Data Collection Centres). Consent was either obtained in writing, via a letter sent by attending NHS clinician, or face-to-face following clinic. For those with ID, the consenting process involved providing ‘easy read’ study information and specially developed consent forms. Where informed consent was not possible, consent could be gained from a family member or appropriate carer.

### 2.2 Data collection and categorisation

All research participants were living in community rather than in-patient settings. All data collected was obtained from review of NHS medical records. Records were reviewed for a period of up to fifteen months. Data were collected at five time points; three months prior to commencement of LCM, date of LCM commencement and then three, six and twelve months post commencement. Endpoint was defined as either one year if the individual continued with LCM treatment or some point within this period if the drug was withdrawn. Demographic data, seizure type, concomitant AEDs, starting and maximum dosage of LCM, length of exposure, adverse effects, dropout rates and seizure frequency were all collected. Standardised case report forms and data collection training were provided remotely to Data Collection Centres where required with data transferred to a pre-defined standardised Excel spreadsheet. Participants were allocated non-identifiable ID numbers.

Duration of epilepsy was ascertained in intervals of five years. Withdrawal rates were estimated as the proportion of people who discontinued LCM within the first year. Seizure frequency was recorded as monthly seizure numbers if evident in medical records, but consolidated into percentage improvement from baseline. Outcomes following treatment were defined therefore as “worsening”, “no improvement” or improvement equivalent to or greater than “25 %”, “50 %” or “75 %+”, with allocation ascertained by calculating difference in recorded seizures numbers at endpoint and baseline. Where seizures were not numerically recorded, frequency and category were determined from clinically recorded impression where appropriate terminology was available.

ID severity was obtained from medical records, with input from GP health profiles or local clinicians where uncertainty existed [22]. Pre-existing health conditions were divided into physical, neurodevelopmental and mental co-morbidities, with mental health further broken down into psychotic and non-psychotic conditions. As with previous AED register arms, side-effect profile for LCM mirrored those detailed in the UK British National Formulary.

### 2.3 Analysis

The Chi-squared test was used to test for univariate associations between outcomes (withdrawal, efficacy, adverse events) and ID group (general population/mild ID/moderate to profound ID) with p-values estimated by Monte Carlo simulation. Differences in tolerance, retention and risk of side-effects between general population patients and ID groups were further explored using logistic regression analysis. Potential sources of confounding bias were addressed through adjustment of regression models for demographic factors and baseline severity.

Differences between ID groups were reported as odds ratios estimated from univariable logistic regression models. Age and gender were added to these models as explanatory factors and the results reported if the adjusted model provided a better fit to the data. The threshold for statistical significance was p = 0.05. Associations with 0.05 p < 0.1 were reported as marginally statistically significant.

## 3 Results

Results are presented in Table 2. Two hundred and thirty two people from 11 UK NHS Trusts (the lead site and 10 Data Collection Centres) consented for data collection from their medical records. Participants were made up of 156 PWE from the general population (88 female) and 76 PWE who also had an ID (40 female). Of this ID group 24 had mild ID (11 female) and 52 had moderate-profound ID (29 female). Age ranged between 18 and 76 years (with a mean age of 53.2 across all participants). Only PWE over 18 years of age were consented. Data were missing for the age of 39 recruits.

**Table 2:** Demographics and clinical features of patients that underwent Lacosamide treatment.

	All Patients	No ID	Mild ID	Moderate – Profound ID
**Age**				
<40	107 (55 %)	56	16	35
40-60	61 (32 %)	49	3	9
60+	25 (13 %)	25	0	0
Missing (39)				
**Gender**				
Male	104 (45 %)	68	13	23
Female	128 (55 %)	88	11	29
**Existing conditions**				
**Physical health**				
Yes	129 (56 %)	88	8	33
No	103 (44 %)	68	16	19
**Mental health (non-psychotic)**				
Yes	60 (26 %)	44	8	8
No	172 (74 %)	112	16	44
**Mental health (psychotic)**				
Yes	4 (2%)	3	0	1
No	228 (98 %)	153	24	51
**Neurodevelopmental**				
Yes	51 (22 %)	8	9	34
No	181 (78 %)	148	15	18
**Lacosamide dose**				
Mean starting dose	80 mg	74	83	94
Mean Max dose	284 mg	283	302	279

The sample size of n = 156 patients without ID and n = 52 patients with moderate to profound ID provides 90 % power at a significance level of 5% to detect a group difference in drop-out rates of 25 %, assuming a rate of 50 % in the non-ID group. Although the study was adequately powered to detect large effect sizes, the study was underpowered to detect small to moderate effect sizes that could still have clinical implications. By pooling together all ID patients, the study would be powered to detect a smaller difference in drop-out rates of 19 %.

Existing physical health conditions were evident in 129 (56 %) of all participants, with similar breakdown marked across different study cohorts (non-ID n88, 56 %, all ID n41, 54 %). Existing non-psychotic mental health conditions were recognised in 60 (26 %) of all people, with psychotic mental health conditions evident in four (2%) and neurodevelopmental conditions apparent in 51 (22 %). Although psychotic and non-psychotic conditions were similarly evenly spread across ID and non-ID cohorts (Table 2.), neurodevelopmental disorders were, as anticipated, far more prominent in the ID population - with nine (36 %) of those with mild ID and 34 (65 %) of those with moderate-profound ID, compared to only eight (5%) of non-ID PWE. 164 out of 196 (84 %) of research participants (36 missing) had a history of epilepsy for over 15 years. This included 38 of 41 individuals with moderate-profound ID, 19 out of 21 with mild ID and 107 out of 134 of those without ID.

Mean starting dose was 80 mg across all recruits (median 50 mg) with mean maximum dose 284 mg (median 300 mg). Test of difference between ID groups were not significant (p = 0.76 for starting dose, p = 0.37 for maximum dose)

Approximately one in five (n43, 20 %) of those for whom withdrawal data was available (n220 of 232) withdrew from LCM during the 12 months since first prescription (Table 3). This was similar for ID (n14 – 20 %) and non-ID groups (n29 -19 %), although mild ID PWE (n2 -10 %) were less likely to withdraw than those with moderate-profound ID (n12 -24 %). The association between withdrawal rate and ID group was not significant (P = 0.31).

**Table 3:** Withdrawal.

	All patients	No ID	Mild ID	Moderate – Profound ID
Missing	12 (5%)	7	3	2
Yes	43 (19 %)	29	2	12
No	177 (76 %)	120	19	38

Limited numbers restrict potential for any inference of statistical significance for reason for withdrawal between ID groups and non-ID patients (Table 4). However when comparing all ID and non-ID patients, lack of efficacy was recorded more often as a reason for withdrawal for ID rather than non-ID patients with over 50 % of ID patient (7 of 13) withdrawing due to lack of efficacy compared to 17 % of non-ID patients (5 of 29). Alternatively, increased seizures were recorded more often for non-ID patients with 31 % for non-ID patients (9 of 29) compared to 15 % for ID patients (2 of 13).

**Table 4:** Reason for withdrawal.

	All patients	No ID	Mild ID	Moderate – Profound ID
Missing	12	7	3	2
No withdrawal	177	120	19	38
Increased seizures	11	9	0	2
Intolerable	14	11	1	2
Lack of efficacy	12	5	1	6
Other	5	4	0	1

There was also no difference in efficacy for those with or without ID (Table 5), with similar recorded data relating to the effect of LCM on people’s seizures (p = 0.93 for association between ID group and efficacy). With missing data removed, greater than 50 % seizure improvement was evident in 43 % (48 of 111) non-ID, 44 % (8 of 18) in the mild ID and 38 % (16 of 42) of moderate-profound ID. Seizure improvement was similar across groups. Efficacy data was missing for 61 participants. This is likely due to minimal or absent recording of seizure frequency data in medical records. Forty four of these individuals remained on the medication with nine withdrawing (withdrawal data was missing for the remaining eight).

**Table 5:** Efficacy.

	All patients	No ID	Mild ID	Moderate – Profound ID
Missing	61 (26 %)	45	6	10
Worsening	27 (12 %)	19	2	6
No change	48 (21 %)	29	6	13
25 % improvement	24 (10 %)	15	2	7
50 % improvement	30 (13 %)	21	2	7
75 % improvement	42 (18 %)	27	6	9

Physical health side-effects were evident in 30 % (n70) of the total cohort, whilst mental health side-effects were evident in only 3% (n 5). As with other findings, prevalence of physical and mental health side-effects was a similar across study cohorts (p = 0.21 and 1.00 respectively for associations with ID group). For the full ID cohort physical health side effects were reported in 22 % (n17) and 4% (n3) PWE (Table 6).

**Table 6:** Side-effects.

	All patients	No ID	Mild ID	Moderate – Profound ID
Physical				
Yes	70 (30 %)	53	5	12
No	162 (70 %)	103	19	40

Mental				
Yes	8 (3%)	5	1	2
No	224 (97 %)	151	23	50

Data were also pooled to compare full ID population (mild ID and Moderate-Profound ID) with the non-ID population for the analysis detailed above. Data comparisons were non-significant apart from the analysis of titration data at different time points (Table 7.). Whilst mean (and median) increases in dose from baseline to the maximum dose are similar for both groups (Non-ID and all ID patients), the difference between dose at baseline and 3 months later was found to be significantly larger in the non-ID population (p = 0.02).

**Table 7:** Titration comparison (pooled ID participants).

	Non-ID	ID patients (all)	p-value
Baseline to 3 months			
Mean	157 mg	123 mg	0.02
Median	150 mg	125 mg	
Baseline to max dose			
Mean	209 mg	196 mg	0.54
Median	200 mg	200 mg	

## 4 Discussion

The research reported in this paper uses the standardised EP ID/PDD AED Register to present a real world observational study of LCM use in PWE from the general population and PWE with ID. Analysis is focussed on studying similarities and differences between and across participant groups with the ID population split into those with moderate-profound ID and those with mild ID. Further analysis comparing all participants with ID and all non-ID participants was undertaken.

The overriding theme within the data reported is the similarities in responses to LCM across the different participant groups. Seizure frequency and intensity (efficacy), withdrawal rates within twelve months and reported side-effects across PWE from the general population and PWE who have mild or moderate-profound ID are comparable. Starting dose and maximum dose of LCM treatment were similar across the different populations. Data reported on the measured metrics were also similar when ID groups were combined and compared with the non-ID group. A major finding is a possible association to high retention rates in the ID population linked to slower titration in the first three months of inception. This provides evidence to what has been of late suspected that people with ID benefit from slower titration of medication than suggested by the drug manufacturers or licencing authorities with the preliminary goal being retaining the drug.

Our data builds on the limited but growing evidence base around the use of LCM with people who have ID. Our methodology is similar to previous research detailed in our introduction [18,4,28]. The presentation of ID and general population data alongside following identical collection methods is a particular strength our study. Despite similarities across the all four studies there are also variations when comparing the data which merit further discussion.

Firstly, the twelve month withdrawal rate for our ID participants is smaller than that reported in the three other studies, with only 20 % withdrawing and 76 % remaining on the study medication (4% missing), compared to a 62 %–64 % retention across other studies [18,4,28]. Interesting the general population cohort in the UK study was also less likely to stay on LCM for 12 months compared to our general population cohort (68 % compared to 77 %). The fact that the UK study reported from a data collection period five years before our study, when LCM was relatively new to the market, is worth noting. Our data reported around titration differences between ID and general population, illustrating a cautious approach to initial LCM prescription in the ID population. Titration data from the other studies have not been reported but are expected to have followed the manufacturers or licencing authorities’ guidance.

The UK study also looked at seizure improvement in a similar way to our study, reporting more than 50 % improvement in 32.7 % of ID PWE, smaller than the 42 % of PWE in our ID cohort. The Dutch study reported undefined ‘seizure reduction’ in 48.5 % while the German study did not report seizure reduction.

There are clearer differences in relation to side effects reported across the studies. The German studies of overall numbers (19 %) are similar to our findings and the rare reports of mental health/behavioural reported within our study mirror those in the UK study. However the UK study reported far more physical health side-effects, whilst the Dutch study reports much higher overall side-effect (63 %) and behavioural side-effects (30 %). The German study talks of high levels of behavioural concerns (31.6 %) but does not explain whether they were baseline or associated with treatment.

Whilst some of the above comparisons are intriguing, the clear differences across participant datasets, implies that any inference particularly of generalizability must be made cautiously. Data were collected for different time periods. Differences in the age of cohorts, patient settings and ID type will have also likely impacted on the data. Factors such as prescriber background, prescribing tendencies and institutionalization care are potential confounders. As with all studies which draw retrospectively from clinical records, the quality of the data reported is also dependent on what is available to be captured across different electronic or paper medical records. Again this may vary with different healthcare systems and cultures.

There are also other specific limitations for our study which should be considered. As detailed above we only collected data from participants who consented to data collection from their medical records. Data Collection Centres were asked to approach all PWE currently or previously prescribed LCM but had varying resources to do this and we do not have data on specific recruitment rates of each centre. It could be argued that those still on LCM may have been more likely to consent to the study and that this may impact on our higher retention rates when compared to the other studies. Lastly, collecting data for PWE and ID is particularly challenging. This is due to the possibility for seizure activity or side-effects being unnoticed or underreported as a result of multiple comorbidities and communication difficulties.

## 5 Conclusions

Despite the limitations detailed above, the findings reported here, and comparisons with broadly similar data from other European retrospective studies of patient medical records, help address the evidence gap around the safe and effective use of LCM within the ID population. Our data suggests that clinicians may expect similar results in people with ID in relation to efficacy, withdrawal, impact on seizures and side-effects when considering prescribing of LCM as with the general population. Our data also suggests that clinicians from our DCCs appear to initially proceed with caution when titrating for LCM within the first three months of prescription and do not increase the dose as rapidly as they do for the general population. Whilst it cannot be concluded that this titration impacts on the high retention in our ID population, its association is a worthwhile clinical point to keep in mind.

## Disclosures

No known conflict of interest for JA, SH, AS, SR, AP, SP, SE, MMo BM has received research grants, honoraria and meeting sponsorship from Eisai, UCB Pharma, Livanova, Bial, GSK, Merck-Serono, Biogen, Novartis, Roche and Sanofi-Genzyme. PT has received honoraria from Bial, Eisai, UCB Pharma and educational support from Veriton. MMa and MB have received honoraria from UCB Pharma, LivaNova and Eisai. RS has received institutional and research support and/or personal fees from LivaNova, UCB, Eisai, Special Products, Bial and Desitin outside the submitted work.

## Funding

This study is supported by a UCB pharma Investigator instigated research grant post independent blinded peer review of the project. UCB Pharma had no role in any study related activity including the study design, recruitment, analysis and report writing. The study has also been adopted by NIHR onto their portfolio.

## Declaration of Competing Interest

No known conflict of interest exists for any of the authors involved in this manuscript.
